# Drug Therapy in the Progressed CML Patient with multi-TKI Failure

**DOI:** 10.4084/MJHID.2015.014

**Published:** 2015-02-15

**Authors:** Ibrahim C. Haznedaroglu

**Affiliations:** Hacettepe University, Faculty of Medicine, Department of Hematology, Ankara, Turkey

## Abstract

The aim of this paper is to outline pharmacotherapy of the ‘third-line management of CML’ (progressive disease course after sequential TKI drugs). Current management of CML with multi-TKI failure is reviewed. TKI (bosutinib, ponatinib, dasatinib, nilotinib) and non-TKI (omacetaxine mepussecinate, IFN or PEG-IFN) drugs are available. The literature search was made in PubMed with particular focus on the clinical trials, recommendations, guidelines and expert opinions, as well as international recommendations. Progressing CML disease with multi-TKI failure should be treated with alloSCT based on the availability of the donor and EBMT transplant risk scores. The TKI and non-TKI drugs shall be used to get best promising (hematological, cytogenetic, molecular) response. During the CP-CML phase of multi-TKI failure, 2nd generation TKIs (nilotinib or dasatinib) should be tried if not previously utilized. Bosutinib and ponatinib (3rd-generation TKIs) should be administered in double- or triple-TKI (imatinib and nilotinib and dasatinib) resistant patients. The presence of T315I mutation at any phase requires ponatinib or omacetaxine mepussecinate therapy before allografting. During the AP/BC-CML phase of multi-TKI failure, the most powerful TKI available (ponatinib or dasatinib if not previously used) together with chemotherapy should be given before alloSCT. Monitoring of CML disease and drug off-target risks (particularly vascular thrombotic events) are vital.

## Introduction

Chronic myeloid leukemia (CML) is a slowly progressive clonal malignant disease characterized by myeloid neoplastic expansion with heterogeneous clinical manifestations.

Tyrosine kinase inhibitors (TKIs) therapy induces high rate of response in the majority of patients. However, while a large proportion of patients attains a prolonged molecular response, and some of them could be considered cured, a not negligible number of patients show a resistance to TKIs therapy.^1^ Standardized therapeutic approach may be useful in the *de novo* or TKI-responsive patient with CML since TKIs could successfully modulate the disease course.^2^–^4^ On the contrary, the treatment schedule should be personalized in the CML patient with progressive disease, despite the administrations of more than one TKI (multi-TKIs).^1^

Disease progression under TKIs is a ‘difficult-to-treat’ situation with the available drugs in CML.^5^

The aim of this paper is to outline the perspectives for the drug therapy choices in the CML patient with progressive disease course after sequential multi-TKIs regimen. This clinical approach is known as ‘third-line management of CML’ in the current TKIs era.

## CML Disease Status and Challenges after multi-TKI Failure

The decision for the choice of TKIs drug depends upon the best available evidence obtained from randomized clinical trials (RCT), physician experience, and characteristics of the patient and his/her disease. In the “real world”, the management of resistant CML should have an integrative approach including: drug (efficacy, safety, tolerability, toxicity, and pharmacoeconomic of the TKI), the patient (CML disease risk, age, co-morbidities, molecular BCR-ABL dynamics, compliance, lifestyle, adherence, drug off-target risk profile), and the status of local medical assistance (TKI availability, TKI reimbursability, drug/disease experience of physician, CML monitoring techniques, the cooperation between CML specialized centre and the home doctors).^1^ Most of those critical parameters are negatively affected in the CML patient with progressive disease course after sequential multi-TKI regimen. TKIs have been investigated in RCT mostly in the newly diagnosed, de novo, first-line patients. Clinical investigations, made in the CML patients where imatinib failed, are mostly open-label, non-comparative trials. Furthermore, the sequential use of 2^nd^ generation TKIs (nilotinib and dasatinib, one after the other) had not been studied in well-designed proper prospective randomized clinical trials.

**Figure 1:**
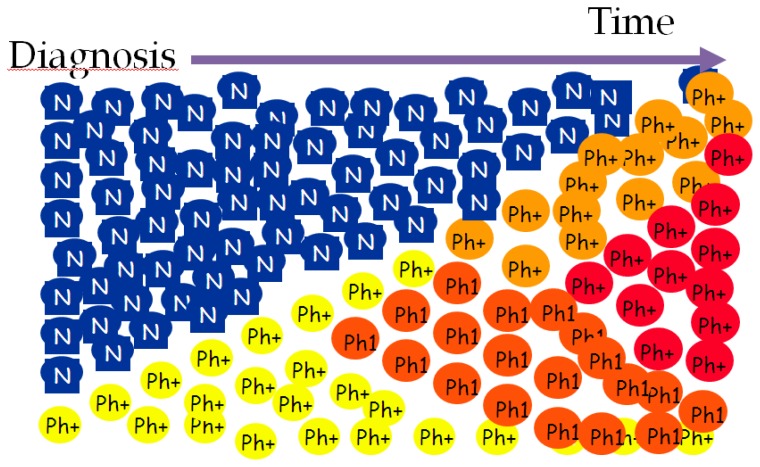
Pathobiological course of Chronic Myeloid Leukemia (CML)* *courtesy of Prof. Giuseppe Saglio

Disease duration is important in the pathobiology of CML. Figure 1 illustrates that time is matter in CML. As a function of time, Ph*(+) neoplastic hematopoiesis dominates blood cell production at onset of the disease. Over time, self-renewal of leukemic CML stem cells, genomic instability, impaired DNA repair mechanisms, proliferation/anti-apoptosis of Ph*(+) neoplastic progenitors, clonal selection, and the acquisition of additional mutagenic events do complicate the biology of CML, as well as the clinical manifestations. Moreover, the oxidative stress, increased by BCR-ABL tyrosine kinase, and the altered mutational phenotype further accelerate the disease course.^5^

Terminal stage of this malignant neoplastic development is the accelerated phase (AP)/blastic crisis (BC) of CML. Late progressing chronic phase (CP), uncontrolled under TKIs, is also a precarious situation prone to AP/BC CML. The terrible end of CML makes ‘prevention of disease progression’ is the ultimate aim of TKI treatment. Thus, early and rapid reduction of BCR-ABL with acceptable TKIs control is a primary goal of CML therapy. Of course, this goal had already failed in the CML patients with progressive disease after the intake of sequential multi-TKI regimens^2^. Most importantly, after each additional failed treatment line, the probability of developing new mutations (including compound mutations that confer high-level resistance to TKI therapy) and CML progression enhance.

## How to Proceed to Manage CML Disease after multi-TKI Failure?

European LeukemiaNet (ELN) recommendations indicated the way of management in the *de novo* or TKI-responsive CML patient, based on the data obtained from numerous RCTs.^2^ However, the level of evidence is low for decision making about the choice of drugs in the CML patient resistant to previous multi-TKI drugs. Official ELN recommendation for third-line CML treatment (failure of and/or intolerance to 2 TKIs) in CP-phase is “.. *Anyone of the remaining TKIs; allogeneic hematopoietic stem cell transplantation (alloSCT) recommended in all eligible patients (HLA type patients and siblings; search for an unrelated stem cell donor; consider alloSCT)*” quite similar to the suggestions in the AP/BC phase-CML “.. *Anyone of the TKIs that were not used before progression (ponatinib in case of T315I mutation), then alloSCT in all patients. Chemotherapy is frequently required to make patients eligible for alloSCT*”.^2^

Several clinical scenarios (and drug suggestions accordingly) can be generated to describe the ‘third-line CML’ from the ELN recommendations;

- The CML patient with failure of imatinib and dasatinib (candidate for nilotinib, bosutinib, ponatinib; then alloSCT)
- The CML patient with failure of imatinib and nilotinib (candidate for dasatinib, bosutinib, ponatinib; then alloSCT)
- The CML patient with failure of nilotinib and dasatinib (candidate for bosutinib, ponatinib; then alloSCT)
- The CML patient with failure of nilotinib and bosutinib (candidate for dasatinib, ponatinib; then alloSCT)
- The CML patient with failure of dasatinib and bosutinib (candidate for nilotinib, ponatinib; then alloSCT)

The timing of alloSCT has changed to third- or fourth-line CML after failure of the second-generation TKIs.^2^,^6^ The definition of transplant eligibility is never absolute since it is based on the balance between the disease risk of CML and the mortality/morbidity risk of alloSCT.^5^

Mutational analyzes shall be performed in all of the CML cases with multi-TKI failure during the drug treatment decision. BCR-ABL1 kinase domain point mutations are detectable in about 50% of patients with treatment failure and progression. The mutations detected during the TKI therapy may be resulted in drug switches based on the nature of the mutation. Dasatinib and nilotinib retain activity against most of the mutations that confer resistance to imatinib. Likewise, distinct mutations exhibit decreased sensitivity to dasatinib versus nilotinib.^7^ T315I, Y253K, E255K, E255V, F359V, F359C, are the mutations poorly sensitive to nilotinib; whereas T315I, T315A, F317L, F317C, V299L are the mutations poorly sensitive to dasatinib. There is also an extensive evidence that Bosutinib al has activity against most of the mutations that confer resistance to imatinib. Bosutinib also showed activity against Nilotinib resistant mutations including Y253H, E255K/V and F359C/V and Dasatinib resistant mutations including F317C and E255K/V. T315I is a unique mutation making the CML patient irresponsive to all available TKIs but ponatinib, non-TKI drug omacetaxine mepussecinate and allografting.^7^ In the CML patients with the T315I mutation, where effective treatment options are limited, ponatinib continued to exhibit deep and durable responses with up to 6 years follow-up. Dose reductions, to manage adverse events, did not impact maintenance of cytogenetic response. The response rate and safety profile of T315I patients were comparable to those observed in the overall population of refractory CML and Ph+, ALL patients in ponatinib clinical trials.^8^ No mutation conferring resistance to Ponatinib, so far, has been identified.^2^,^9^,^10^ With longer follow-up and the availability of second and third generation TKIs, most clinically relevant ABL kinase mutations respond to change in TKI therapy following imatinib failure, with the majority of patients achieving durable cytogenetic and molecular responses. An early detection and characterization of ABL kinase mutations shall be performed in imatinib-resistant patients in order to identify the patients who may benefit from alternative TKI therapy or stem cell transplantation. In the Palani study^11^, eighty-three ABL kinase mutations were detected in 65 CP-CML patients at the time of imatinib failure with 35% of patients (23 of 65) harboring P-loop mutations (including M244V), 18% (12 of 65) with T315I mutation and 46% (30 of 65) with other mutations (catalytic domain, imatinib binding site, activation loop and C-terminal). Composite mutations were present in 10 patients (15%), with two patients harboring both P-loop and T315I mutations.^11^

## Difficulties in the Treatment of CML after multi-TKI Failure

The most challenging situations are failure to all available TKIs in the patients CML which cannot be transplanted or relapsing after allografting. These patients need effective and safer treatment options. Therefore, patient-centered clinical decision is necessary in this difficult situation.^5^ Table 1 summarizes the current status of ‘third-line management of CML’ with the available drugs. Before the introduction of bosutinib and ponatinib, CP-CML patients who had failed 2 prior lines of TKI had limited treatment options with poor treatment response and outcome. These newer TKIs are important additions to the treatment armamentarium, but the optimal choice of 3rd-line CML treatment has not been established.^12^

Bosutinib is an oral dual Src/Abl TKI drug. It is recommended in 2nd, 3rd and later lines of CML treatment in both ELN and NCCN guidelines, where appropriate.^2^ This is based on the large 2nd line bosutinib study and 3rd line study with 119 patients – only a few patients were “triple TKI failed” patients. In this last study, the CML patients (n=119) were aged ≥18 y, with prior imatinib failure plus dasatinib resistance (n=38) or intolerance (n=50), nilotinib resistance (n=26), or nilotinib intolerance or dasatinib resistance/intolerance + nilotinib resistance/intolerance (n=5) and received bosutinib starting at 500 mg/day.^13^ Major cytogenetic response (MCyR) was newly attained or maintained from baseline by 33% and 7% of patients, respectively (32% attained/maintained complete cytogenetic response [CCyR]). Kaplan-Meier probability of maintaining MCyR or CCyR at four y was 69% and 54%, respectively. The most common adverse event with bosutinib was diarrhea (n=98).^13^ Therefore, to suggest that Bosutinib can only be administered in triple TKIs failed patients after imatinib, nilotinib and dasatinib does not reflect the wealth of the data nor the recommendations. This also fits with the patient centred approach^1^ which suggests that based on co-morbidities, disease risk, and mutations. The choice in 3rd line can be from among nilotinib, dasatinib, bosutinib and ponatinib rather than stipulating the order that they must be used. Bosutinib could be an option for the CML situations which preclude the use of other TKIs.^14^ In a Spanish study, cross intolerance with bosutinib was extremely rare, of the 7 patients who had rash with imatinib, only 1 suffered rash with bosutinib. None of the patients had pleural effusion with bosutinib out of 15 who previously experience it with dasatinib neither vascular events out of the ten patients that already had this side effect with nilotinib. Therefore, in heavily pretreated CML patients, most of them in 4th-line, bosutinib had an acceptable safety with no CML patients interrupting treatment due to side effects also in the previously TKI intolerant patients. Importantly, the rates of cross intolerance (namely cardiovascular, pleural and skin) were also very low in the Spanish study.^14^

Ponatinib is an approved potent oral TKI active drug against native and the mutant BCR-ABL, including the resistant T315I mutant. The PACE trial evaluated the efficacy and safety of ponatinib (45mg qd) in CML and Ph+ ALL patients (n=449) resistant/intolerant to dasatinib or nilotinib or with the T315I mutation.^15^ Ponatinib is effective in 67% of CP-CML third-line patients. In the PACE Study, 98 patients received ponatinib in 3^rd^ line (after failure of imatinib and dasatinib or imatinib and nilotinib. In this subgroup, the response rate (MCyR) was 67% comparable to that achieved with dasatinib and nilotinib in earlier treatment lines (second line). In both ELN and NCCN guidelines, Ponatinib is recommended for use in 3^rd^ line; with the ELN guidelines also indicating ponatinib for use also in 2^nd^ line. Ponatinib is the only single TKI, which has been extensively studied in a large cohort of patients where imatinib and a 2^nd^ generation TKI have failed. On the contrary, sequential use of nilotinib and dasatinib in third line has not been studied in appropriately designed clinical trials and the scarce available published evidence suggests a scarce efficacy. Garg and co-workers indicated that the use of second-generation TKI after failure to two TKIs may induce clinical responses, but those are usually not durable except in some CP-CML patients.^16^ Likewise, bosutinib give a modest benefit in 3^rd^-line CML^17^. Only 32% of the third-line patients achieved MCyR, and this despite that close to half (46%) of the patients has not had a problem of resistance at baseline, but was intolerant to previous TKIS.^17^ The poor efficacy and short duration of response in patients treated with nilotinib and dasatinib in sequential use, highlight that one reason for the lack of durable cytogenetic remission could be the emergence of new kinase domain mutations. Ponatinib could provide a higher probability of response for patients failing imatinib and dasatinib/nilotinib compared with sequential 2nd generation TKI therapy commonly used in this indication.^18^ In the patients, where one 2^nd^ generation TKI has failed, the risk of disease progression is high, and ponatinib, which has demonstrated an unprecedented efficacy in difficult-to-treat patient population, may be considered as the therapeutic option, even if it presents an increased risk of thrombotic vessel occlusions. In fact, in October 2013, PACE trial was placed on partial clinical hold, due to observation of arterial thrombotic events in the ponatinib clinical program; following these events, a dose reduction was recommended. Serious arterial thrombotic episodes were observed in 19% of the ponatinib-treated patients and included cardiovascular 10%, cerebrovascular 7%, peripheral vascular 7% districts. Venous thromboembolic events too were signaled in 5% of the ponatinib-treated patients. Higher dose-intensity, older age, and cardiovascular risk factors were associated with higher likelihood of thrombotic events. However, Ponatinib could exhibit deep and durable responses in heavily pre-treated patients (58% received ≥3 prior TKIs) with relatively longer follow-up (median follow-up: 27.9 (0.1–39.5) months), particularly CP-CML. Initial data suggested that the response may be maintained after the dose reduction; however, longer follow-up is needed to understand impact on safety.^15^ In the refractory CML patients, the rapid and profound reduction in BCR-ABL levels, achieved with Ponatinib, translated into improved long-term outcomes. The assessing BCR-ABL levels at early time points, as a goal of therapy with Ponatinib, had been suggested since achieving early landmark response could be a reliable predictor of better long-term outcomes.^19^ In an ongoing phase 1/2, multi-center, open-label, dose-finding study of ponatinib in Japanese patients with CML or Ph+ ALL, who have experienced a failure of dasatinib or nilotinib therapy, because of resistance or intolerance, ten (59%) CP-CML patients attained the primary efficacy endpoint of MCyR (6 CCyR, 4 MMR). The primary efficacy endpoint of major hematological response was achieved by 10 patients: 2/2 AP-CML, 2/4 BP-CML and 6/12 Ph+ ALL patients. MMR was observed in 5 (14%) CML patients in the Japanese study.^20^ Ponatinib dose intensity is associated frequently with many adverse side effects. Future investigations (a dose-ranging trial of ponatinib in refractory CML to evaluate benefit/risk of different dosing schemes) should focus on lower average ponatinib dose intensity, such as starting at lower doses and/or reducing the dose basing on the response level in CML.^21^ Real-world data also indicated that ponatinib is prescribed across disease phase, therapy line, and mutation status. Physicians have adopted dose-reduction strategies in both new and especially ongoing patients; evidence indicates dose-adjustment by age, gender and disease phase.^22^

Clinical trials in patients who have failed 2 previous lines of TKI suggest ponatinib may be more efficacious than bosutinib, but with a less favorable side-effect profile.^12^,^17^ The comparison of overall benefit-risk from available clinical trial data is challenging due to single- arm designs, low overall mortality, disparate impact of characteristic side-effects, and the likely crossover/sequential use of alternative TKIs among patients discontinuing therapy.^12^ Levy and coworkers examined the efficacy outcomes, treatment duration and reason for study drug discontinuation, as surrogates for overall benefit-risk in 3rd line CP-CML patients treated with ponatinib vs. bosutinib.^12^ They used the clinical trial data for bosutinib^17^ and PACE for ponatinib^15^ for 3rd line CP-CML. The study examined efficacy outcomes including MCyR, CCyR, MMR, durability of response, duration on therapy and reasons for discontinuation among patients treated with ponatinib vs. bosutinib after failing 2 prior TKIs. In the study, the outcomes were evaluated at similar follow-up time points: median 28.5 (range 0.3–56.2) months bosutinib; median 30.5 (0.2–39.8) months ponatinib. The efficacy outcomes were defined such that patients were required to demonstrate improvement relative to baseline to be counted as responders.^12^ Their indirect comparison using a variety of surrogate measures suggested superior efficacy and durability of response with ponatinib vs. bosutinib in 3rd line CP-CML patients. Based on the results of this indirect comparison, the treatment response was higher for 3rd line CP-CML patients treated with ponatinib (n=98) than with bosutinib (n=118) across all measures. MCyR was achieved by 67% of ponatinib vs. 32% of bosutinib patients, CCyR by 56% of ponatinib vs. 24% of bosutinib patients, and MMR by 42% vs. 15%. The CML patients who received ponatinib experienced more durable responses with 93% of the ponatinib patients who achieved MCyR estimated to retain response after 2 years vs. 59% of the bosutinib patients who achieved this response level. After approximately 2.5 years of follow up, less than one-third (29%) of bosutinib patients remained on study drug vs. the majority (57%) of ponatinib patients. The median treatment duration was substantially shorter for bosutinib vs. ponatinib, with patients remaining on ponatinib therapy more than 3.5 times as long as on bosutinib. The majority of 3rd line bosutinib patients that discontinued did so due to treatment failure (58.3% of the patients who discontinued), while less than one-quarter (23.8%) of ponatinib patients who discontinued did so due to failure.^12^

## Perspectives for the Treatment of Progressing CML disease after multi-TKI Failure

Current standard practice is allografting for all of the CML cases with multi-TKI failure based on the availability of the donor and EBMT transplant risk scores. Before the alloSCT, all patients should be treated with the best available ‘remaining’ TKI in order to reach best promising response/remission land (complete hematological response (CHR), complete cytogenetic response (CCyR), stable molecular response (MR)). For this aim all the drugs “Remaining”, bosutinib, ponatinib, dasatinib, nilotinib, and omacetaxine mepussecinate should be used. During the CP-CML phase of multi-TKI failure, 2^nd^ generation TKIs (nilotinib or dasatinib) are used if remaining. Bosutinib and ponatinib (3^rd^-generation TKIs) can be administered in double- or triple-TKI failed (Imatinib and nilotinib and dasatinib) patients. The presence of T315I mutation at any phase requires ponatinib or omacetaxine mepussecinate therapy before allografting. Combinations of TKI and interferon (IFN) or PEG-IFN are used in the everyday clinical practice for the unresponsive cases to TKI alone, but limited data is available for the combination approach.^29^,^30^ During the AP/BC -CML phase of multi-TKI failure, the most powerful TKI available (ponatinib or dasatinib if remaining) together with multi-agent chemotherapy^31^ should be given before alloSCT.^32^ The clinical outcome is more poor for the transplant-ineligible CML patients with multi-TKI failure or post-transplant relapsed patients. TKI (bosutinib, ponatinib, dasatinib, nilotinib) and non-TKI (omacetaxine mepussecinate, IFN or PEG-IFN; including their combinations with TKIs) drugs should be used based on the same principles in those problematic CML patients as summarized above. Monitoring the CML disease and drug off-target risks (particularly vascular thrombotic events) are vital. Expected hematological, cytogenetic, and molecular responses to those drugs during the monitoring of CML are variable, and based on the disease phase, mutational status, resistance profile, age, co-morbidities, molecular BCR-ABL dynamics, compliance, lifestyle, adherence, and drug off-target risk profile.^1^ Future candidate CML treatment regimens can be optimized for maximal specificity toward primitive leukemia stem cells.^33^

## Figures and Tables

**Table 1:** Strengths and limitations of the drugs for the ‘third-line’ management of chronic myeloid leukemia (CML)

Drug	Pharmacobiology	Patient population	Efficacy data	Safety, tolerability, toxicity	Clinical challenges
*Ponatinib*	Pan-BCR-ABL kinase inhibitor	Multi-TKI (imatinib, nilotinib, dasatinib) resistant CML patient T315I mutation AP/BC- CML	Major cytogenetic response (MCyR) within the first 12 months in over half of patients with CP- CML and major hematological responses within the first 6 months in at least 50 % of adults with AP- CML and 34 % of patients with BC-CML or Ph*+ ALL after a median follow-up duration of 15, 16 and 6 months, respectively.^23^	The analyses about the 24 months follow up safety data of the PACE trial disclosed non-serious and serious arterial and venous adverse events combined occurred in about 20% of ponatinib-treated patients (Cardiovascular events 6.2%; Cerebrovascular events 4.0%; Peripheral vascular events 3.6%; venous occlusion 2.9%)^24^	Problems of availability and reimbursability^25^ Cost^25^ Thrombotic cardiovascular and cerebrovascular adverse effects^24^
*Bosutinib*	3^rd^ generation dual SRC/ABL TKI	Multi-TKI (imatinib, nilotinib, dasatinib) resistant CML patient	MCyR was attained by 32% of patients; CCyR was attained by 24%, including in one of 3 patients treated with 3 prior TKIs. CHR was achieved/maintained in 73% of patients.^17^	Gastrointestinal adverse effects (diarrhea [86%], nausea [46%], vomiting [37%]). Grade 3/4 myelosuppression [ 41%]. Alanine aminotransferase elevation [17% ]^26^	Problems of availability and reimbursability^25^ Cost^25^ Gastrointestin al comorbidity^2^,^5^
*Omacetaxine mepussecinate*	Induction of apoptosis, non-TKI antiproliferative effect	Multi-TKI (imatinib, nilotinib, dasatinib) resistant CML patient T315I mutation	Forty-six patients were enrolled: all had received imatinib, 83% had received dasatinib, and 57% nilotinib. A median 4.5 cycles of omacetaxine were administered (range, 1–36). CHR was achieved or maintained in 31 patients (67%); median response duration was 7.0 months. Ten patients (22%) achieved MCyR, including 2 (4%) CCyR. Median progression-free survival was 7.0 months [95% confidence interval (CI), 5.9–8.9 months], and overall survival was 30.1 months.^27^	Grade 3/4 hematologic toxicity included thrombocytopenia (54%), neutropenia (48%), and anemia (33%). Nonhematologic adverse events were predominantly grade 1/2 and included diarrhea (44%), nausea (30%), fatigue (24%), pyrexia (20%), headache (20%), and asthenia (20%). 27	Problems of availability and reimbursability^25^ Cost^25^
*Nilotinib*	2^nd^ generation BCR- ABL inhibitor	‘Remaining TKI’ after the failure of imatinib and dasatinib	CHR and MCyR rates in CP were 79% and 43%, respectively. Of 17 evaluable patients with CML-AP, 5 (29%) had a confirmed hematological response and 2 (12%) a MCyR. At 18 months 59% of patients were progression- free.^28^	Rash (28% CP, 19% AP), nausea (15% CP, 10% AP), pruritus (15% CP, 10% AP), headache (13% CP, 5% AP) and fatigue (10% CP, 10% AP). neutropenia (23% CP, 33% AP) thrombocytopenia (28% CP, 19% AP). hyperphosphatemia (13% CP, 24% AP), elevated total bilirubin levels (8% CP, 14% AP), elevated lipase levels (25% CP, 10% AP), hypokalemia (5% CP, 10% AP), hyperglycemia (13% CP, 5% AP), hypermagnesemia (11% CP, 11% AP) 28	Cost^25^ Pancreatic and metabolic comorbidity^2^,^5^
*Dasatinib*	2^nd^ generation BCR- ABL and SRC inhibitor	‘Remaining TKI’ after the failure of imatinib and nilotinib	Among the 14 patients treated with dasatinib as second-line treatment, 8 patients were in CP (57%), 3 in AP (21%), and 3 in BP (21%). The best response to dasatinib included 2 CCyR (14%), 1 PCyR (7%), 5 mCyR (36%), 4 CHR (29%), and 2 NR (14%).^16^	7 patients (21%) discontinued treatment because of toxicity despite an acceptable response, including 2 patients who discontinued because of pleural effusion, and 1 each for gastrointestinal bleeding, neutropenia, renal failure, atrial fibrillation, and myalgias.^16^	Cost^25^ Lung comorbidity^2^,^5^

## References

[1] Haznedaroglu IC (2013). Current concerns of undertreatment and overtreatment in chronic myeloid leukemia based on European LeukemiaNet 2013 recommendations. Expert opinion on pharmacotherapy.

[2] Baccarani M, Deininger MW, Rosti G (2013). European LeukemiaNet recommendations for the management of chronic myeloid leukemia: 2013. Blood.

[3] Lipton JH, Chuah C, Guerci-Bresler A (2014). Epic: A Phase 3 Trial of Ponatinib Compared with Imatinib in Patients with Newly Diagnosed Chronic Myeloid Leukemia in Chronic Phase (CP-CML). Blood.

[4] Baccarani M, Castagnetti F, Gugliotta G, Palandri F, Rosti G (2014). Treatment Recommendations for Chronic Myeloid Leukemia. Mediterr J Hematol Infect Dis.

[5] Zhaleiko IO, Perekhrestenko TP, Bilko DI, Dyagil IS, Bilko NM (2014). Determination of the optimal chemotherapy drugs pretreatment time through cultivation of hemopoietic cells in CML-patients treated with tyrosine kinase inhibitors. Exp Oncol.

[6] Uz B, Bektas O, Eliacik E (2011). Allografting for Bosutinib, Imatinib, Nilotinib, Dasatinib, and Interferon Resistant Chronic Myeloid Leukemia without ABL Kinase Mutation. Case reports in hematology.

[7] Mathisen MS, Kantarjian HM, Cortes J, Jabbour EJ (2014). Practical issues surrounding the explosion of tyrosine kinase inhibitors for the management of chronic myeloid leukemia. Blood reviews.

[8] Mauro MJ, Cortes JE, Hochhaus A (2014). Ponatinib Efficacy and Safety in Patients with the T315I Mutation: Long-Term Follow-up of Phase 1 and Phase 2 (PACE) Trials. Blood.

[9] Haznedaroglu IC (2014). Monitoring the Response to Tyrosine Kinase Inhibitor (TKI) Treatment in Chronic Myeloid Leukemia (CML). Mediterr J Hematol Infect Dis.

[10] Miller GD, Bruno BJ, Lim CS (2014). Resistant mutations in CML and Ph(+)ALL - role of ponatinib. Biologics: targets & therapy.

[11] Palani R, Szydlo RM, Apperley JF (2014). Clinical Outcome Following Change of Tyrosine Kinase Inhibitor (TKI) According to the Detection of an ABL Kinase Mutation. Blood.

[12] Levy MY, McGarry LJ, Huang H, Lustgarten S, Nieset C, Haluska FG (2014). Benefit-Risk of Ponatinib Vs. Bosutinib in Chronic Phase Chronic Myeloid Leukemia (CP-CML) Patients Who Failed Two Prior Tyrosine Kinase Inhibitors (TKIs): An Indirect Comparison. Blood.

[13] Gambacorti-Passerini C, Khoury HJ, Kantarjian HM (2014). Bosutinib As Third-Line Therapy in Patients (Pts) with Chronic Phase Chronic Myeloid Leukemia (CP CML) Following Failure with Imatinib Plus Dasatinib and/or Nilotinib: 48-Month Update of a Phase 1/2 Study. Blood.

[14] García-Gutiérrez V, Maestro B, Martinez-Trillo A (2014). Bosutinib Apears to be Safe, with Low Cross Intolerance, in Patients Treated in 4th Line. Results of the Spanish Compassionate Use Program. Blood.

[15] Cortes JE, Kim D-W, Pinilla-Ibarz J (2014). Long-Term Follow-up of Ponatinib Efficacy and Safety in the Phase 2 PACE Trial. Blood.

[16] Garg RJ, Kantarjian H, O’Brien S (2009). The use of nilotinib or dasatinib after failure to 2 prior tyrosine kinase inhibitors: long-term follow-up. Blood.

[17] Khoury HJ, Cortes JE, Kantarjian HM (2012). Bosutinib is active in chronic phase chronic myeloid leukemia after imatinib and dasatinib and/or nilotinib therapy failure. Blood.

[18] Lipton JH, Shah D, Tongbram V (2014). Comparative Efficacy Among 3rd Line Post-Imatinib Chronic Phase-Chronic Myeloid Leukemia (CP-CML) Patients after Failure of Dasatinib or Nilotinib Tyrosine Kinase Inhibitors. Blood.

[19] Mueller MC, Baccarani M, Deininger MW (2014). Achieving Early Landmark Response Is Predictive of Outcomes in Heavily Pretreated Patients with Chronic Phase Chronic Myeloid Leukemia (CP-CML) Treated with Ponatinib. Blood.

[20] Kyo T, Tojo A, Yamamoto K (2014). Ponatinib Safety and Efficacy in Japanese Patients with Philadelphia Positive Leukemia: Update of a Phase 1/2 Study. Blood.

[21] Knickerbocker R, Dorer DJ, Haluska FG (2014). Impact of Dose Intensity of Ponatinib on Selected Adverse Events: Multivariate Analyses from a Pooled Population of Clinical Trial Patients. Blood.

[22] McGarry LJ, Wolfe G, Steagall A, Huang H (2014). Real-World Prescribing Patterns with Ponatinib Among New and Ongoing US Patients. Blood.

[23] Hoy SM (2014). Ponatinib: a review of its use in adults with chronic myeloid leukaemia or Philadelphia chromosome-positive acute lymphoblastic leukaemia. Drugs.

[24] Groarke JD, Cheng S, Moslehi J (2013). Cancer-Drug Discovery and Cardiovascular Surveillance. New England Journal of Medicine.

[25] CML Ei (2013). The price of drugs for chronic myeloid leukemia (CML) is a reflection of the unsustainable prices of cancer drugs: from the perspective of a large group of CML experts. Blood.

[26] Kantarjian HM, Cortes JE, Kim DW (2014). Bosutinib safety and management of toxicity in leukemia patients with resistance or intolerance to imatinib and other tyrosine kinase inhibitors. Blood.

[27] Cortes J, Digumarti R, Parikh PM (2013). Phase 2 study of subcutaneous omacetaxine mepesuccinate for chronic-phase chronic myeloid leukemia patients resistant to or intolerant of tyrosine kinase inhibitors. American journal of hematology.

[28] Giles FJ, Abruzzese E, Rosti G (2010). Nilotinib is active in chronic and accelerated phase chronic myeloid leukemia following failure of imatinib and dasatinib therapy. Leukemia.

[29] Guilhot F, Rigal-Huguet F, Guilhot J (2014). Long Term Outcome of Chronic Phase Chronic Myeloid Leukemia (CP CML) Patients (pts) from the French Spirit Study Comparing Imatinib (IM) 400 Mg to Higher Dose Imatinib or Combination with Peg-interferona2a (PegIFN) or Cytarabine (Ara-C). Blood.

[30] Hanfstein B, Lauseker M, Hehlmann R (2014). Comparing the Prognostic Significance of Early Predictors of Survival in Chronic Myeloid Leukemia (CML) Treated with Imatinib - an Analysis of the Randomized CML-Study IV. Blood.

[31] Romanova E, Girshova L, Siordia N (2014). “FLAG” Regimen Induces Deep, Although Short-Term, Responses in Patients Resistant to Tyrosine Kinases Inhibitors in BP CML”. Blood.

[32] Kim DDH, Bence-Bruckler I, Hillis CM, Walker I, Kamel-Reid S, Lipton JH (2014). Retrospective Analysis of Allogeneic Transplant Outcomes in Chronic Myeloid Leukemia Patients with Tyrosine Kinase Inhibitors Failure. Blood.

[33] Neering SJ, Bushnell T, Sozer S (2007). Leukemia stem cells in a genetically defined murine model of blast-crisis CML. Blood.

